# How do older patients with advanced kidney disease, and their family members, understand kidney function and failure? A qualitative study

**DOI:** 10.1186/s12882-025-04541-1

**Published:** 2025-11-04

**Authors:** Charlotte M. Snead, Robert A. Kimmitt, Fergus J. Caskey, Jocelyn Darling, Leila Rooshenas, Joanna Coast, Rachael L. Morton, Lucy E. Selman, Barnaby Hole

**Affiliations:** 1 Population Health Sciences, Bristol Medical School, University of Bristol, Canynge Hall, 39 Whatley Road, Bristol, BS8 1UD UK; 2 Exeter Kidney Unit, Royal Devon University Healthcare NHS Foundation Trust, Exeter, UK; 3 Department of Clinical and Biomedical Sciences, University of Exeter, Exeter, UK; 4 Patient and Public Involvement (PPI) Contributor, Bath, UK; 5 Richard Bright Unit, North Bristol NHS Trust, Bristol, UK; 6 NHMRC Clinical Trials Centre, The University of Sydney, Camperdown, Australia

**Keywords:** Age, Renal insufficiency, Kidney failure, chronic, Chronic kidney disease, Conservative kidney management, Decision making, shared, Dialysis, Multimorbidity

## Abstract

**Background:**

Patients approaching kidney failure are increasingly older, and living with multiple long-term conditions. The benefits of kidney replacement therapy (KRT) are uncertain for many in this group. Supporting decisions between treatment options requires consideration of how people perceive chronic kidney disease and its treatments. This qualitative study aimed to explore how older patients and family members understand kidney function and failure, and how this impacts treatment decision-making.

**Methods:**

Between September 2018 and July 2019, semi-structured interviews were conducted with older patients and family members recruited from three United Kingdom kidney units. Eligible patients had estimated glomerular filtration rate (eGFR) < 15 ml/min/1.73m^2^, no previous KRT and were age ≥ 80 years, or ≥ 65 years with significant comorbidity. Interviews used a topic guide developed with patient input. Interview transcripts were analysed using inductive thematic analysis and constant comparative techniques.

**Results:**

Fifteen patients and 12 family members were interviewed. Three themes were identified: (i) Critical blood-cleaning organs, where kidney function was considered vital for survival; (ii) Unwitnessed function and failure, where kidney disease was experienced invisibly; and (iii) Quantifying and predicting kidney function, including conceptualisation of kidney function using numbers and graphs. Unwitnessed, intangible experiences of kidney failure appeared to accentuate reliance on clinicians for disease information. Numerical and graphical depictions of kidney function were central to formation of disease understanding. Concepts of treatment ‘thresholds’ appeared to affirm misperceptions of a binary choice between dialysis and death.

**Conclusions:**

Unintended misunderstandings, including eGFR thresholds for dialysis initiation, arise from common clinical communication approaches and appear to impact upon informed decision-making. This is especially important for older patients with multiple conditions, for whom the benefits of dialysis are uncertain. Improved consultation approaches which enhance patient understanding are needed. Revising misleading terminology and shifting focus away from numerical disease metrics may help patients and families making individualised choices between treatments.

**Clinical trial number:**

Not applicable.

**Supplementary Information:**

The online version contains supplementary material available at 10.1186/s12882-025-04541-1.

## Background

Across high-income countries, an increasingly high proportion of people living with chronic kidney disease (CKD) and commencing kidney replacement therapy (KRT) are older, and living with multiple long-term conditions (MLTC) [1]. Less than 1% of this population receive transplants [2] and most receive in-centre haemodialysis [1], despite availability of home treatments. An alternative to KRT is conservative kidney management (CKM), where dialysis is neither planned for nor initiated, but care focuses on prevention of, and relief from, pain and other physical, psychosocial, and spiritual problems [3]. The survival and quality of life benefits of dialysis versus CKM for older people with MLTC remain uncertain [4], with a randomised controlled trial nearing completion in this area [5]. 

Patients and their families require support and information to make preference-sensitive decisions between KRT and CKM [6, 7]. Shared decision-making (SDM), a model involving proactive engagement of patients, healthcare professionals, close family and/or caregivers, is recommended [8], the effectiveness of which requires appropriate education and communication strategies [6, 8]. However the experience of SDM by older adults with CKD can be sub-optimal [9]. 

Qualitative research is crucial for understanding social phenomena such as treatment decision-making, particularly when little prior evidence exists [10]. In-depth qualitative interview studies have shown that individuals preparing for or receiving treatment for kidney failure often misunderstand their disease and its treatments, have knowledge gaps [7, 11–14], and/or are not always aware they have a treatment choice [13–15]. Existing studies have mainly focused on the perspectives of those who have already started dialysis or CKM, on decisions between haemodialysis (HD) and peritoneal dialysis (PD), and often under-represent older patients [11, 16]. To our knowledge, no study has specifically explored how understanding of CKD by older patients and their close family members affects treatment decision-making. We aimed to explore understandings of kidney function, disease progression, and failure in this group, to inform person-centred care.

## Methods

### Study design

This secondary qualitative analysis uses a social constructionist design and is part of a larger study (UNPACK) examining treatment preferences of older patients and their families deciding between dialysis and CKM. The data presented in this report represent one of several outputs from UNPACK which has reported both qualitative [17, 18] and quantitative [19] findings. Reporting is in accordance with the Consolidated Criteria for Reporting Qualitative research (COREQ) [20] [Supplementary file S1].

### Inclusion and exclusion criteria

Patient eligibility criteria were chosen to reflect current evidence concerning the population for whom the comparative benefits of dialysis versus CKM are unclear [4]. Inclusion and exclusion criteria were as follows.

#### Patient participants

Inclusion criteria:

- English-speaking;
- In receipt of specialist nephrology care with estimated glomerular filtration rate (eGFR by the CKD-EPI Eq. [21]) < 15 ml/min/1.73m^2^ on at least one occasion over the preceding 12 months;
- Aged ≥ 80 years irrespective of comorbidity, or ≥ 65 years if they had either two or more additional major long-term conditions [22] or were confined to a bed or chair more than 50% of waking hours [23].

Exclusion criteria:

- Having ever received outpatient KRT.
- Due to start KRT within the next month.
- Unable to provide informed consent.

#### Family member participants

Inclusion criteria:

- English-speaking;
- Aged 18 years or older;
- Nominated by eligible patients as a close individual whom they would involve in decisions about their health.

### Sampling and recruitment

Patients were recruited from three English kidney units. Two were transplant centres, each providing care to approximately 600 dialysis recipients (Sites A and B). One was a non-transplant centre caring for approximately 200 dialysis recipients (Site C). One larger centre (Site B) had a long-established dedicated CKM clinic and multidisciplinary team, whereas the other centres delivered CKM within general nephrology care. Local teams assessed eligibility and provided potential participants with written information materials. The study team invited participation and arranged interviews by telephone. Patients were purposively sampled to maximise clinicodemographic variation. Patients were asked to invite up to two “*family members or friends who help with decisions about your health*”, who opted-in by contacting the study team.

### Data collection

Semi-structured, in-person interviews were conducted between September 2018 and July 2019 in participants’ homes by BH, a white, male, trainee kidney specialist in his late-30s. This was BH’s first experience of qualitative research, conducted as part of his PhD. He had received formal training in qualitative research and interviewing skills. Unless directly asked (this occurred once), he did not disclose his medical training, describing himself as a “researcher”. Socio-demographic information was collected. Where they agreed, participants were interviewed individually. A single semi-structured interview was conducted per participant, using open-ended questioning and supported by a topic guide [previously published [17]; see also Supplementary file S2] developed and piloted based on the literature and with patient input. Interview technique was refined through self-reflective notes and feedback from PhD supervisors at regular meetings. The topic guide and questions were adapted and refined to explore concepts and patterns in the data identified in earlier interviews and initial analysis. Interviews, conducted with only the interviewer and interviewee(s) present, were audio-recorded using an encrypted digital voice recorder, and handwritten field notes were taken. Participants received a £20 voucher for participating. Recordings were transcribed verbatim and managed with QSR NVivo 11 software.

### Patient and public involvement (PPI)

Patients and members of the public were involved in this research at every stage of the primary study, contributing to its conception, design and interview topic guide developed, as previously described [17, 18]. PPI co-author (JD) contributed to critical analysis of the over-arching themes, manuscript review and revisions.

### Methodological and theoretical approach

This study is based on a constructionist framework, drawing from a critical realist ontology and subjectivist epistemology [24]. Critical realism, developed by Bhaskar, suggests that underlying structures exist independently of our awareness, even if they cannot be fully known [25]. Subjectivism holds that knowledge is shaped by social and cultural filters such as language, class, gender, and ethnicity. From this view, knowledge is inherently value-laden [26]. 

### Analysis

Interview transcripts were analysed inductively, using thematic analysis and constant comparative techniques [27, 28]. Braun and Clarke’s six-stepped approach was followed, which involves; (1) familiarisation with the data; (2) initial coding; (3) searching for themes; (4) reviewing themes; (5) defining themes; and (6) producing the report [27]. Transcripts were initially coded line-by-line, independently, by BH, CS and RK, starting with ‘open’ coding to identify concepts and meanings from participants’ views and experiences. Interviews and codes were discussed and compared among the study investigators, with disagreement resolved by discussion, and consideration of wider meaning, alongside reorganisation, recoding and thematic development [17, 19]. A thematic map was developed linking sub-themes into broader overarching parent themes [Supplementary file S3]. Analysis and recruitment were conducted in parallel, with recruitment discontinued when no new themes for the primary analysis were identified [17]. Informational power for the primary analysis was determined through iterative rounds of coding development in conjunction with written analytical accounts, with feedback from the PhD supervisory team.

### Rigour and reflexivity

During data collection, BH kept detailed field and reflective notes. During the triple line-by-line coding of interview data, and analytical account development, CS, RK and BH made reflexive notes. Reflexivity was discussed alongside thematic development during research meetings. Clear documentation was kept of analysis stages, and the presented analysis is supported by a range of excerpts including examples of contrasting excerpts where relevant.

### Ethical considerations

The study was reviewed by Surrey Research Ethics Committee (NHS Health Research Authority and Health and Care Research Wales approval reference 18/LO/1179) and was conducted in accordance with the Declaration of Helsinki. Written informed consent was obtained for all participants.

## Results

Of 33 patients approached, 15 (46%) participated. Ten (67%) patients recruited one or more family members. Overall, 27 participants (15 patients and 12 family members) were interviewed (three patients from Site A, six from Site B, and six from Site C) (Table 1). Reasons for non-participation are detailed in Supplementary file S4. Patient participants were between 68 and 90 years old. All were retired. Gender was balanced, all described their ethnicity as white British, and eGFR ranged from 7 to 14 ml/min/1.73m^2^, excepting one participant whose eGFR was 25 ml/min/1.73m^2^ at the time of interview, having been 14 ml/min/1.73m^2^ at recruitment. Seven patients were preparing for HD, two for PD and the remaining six for CKM; with nobody active on a transplant waiting list.

**Table 1:** Participant characteristics (*n* = 27)

Characteristic	Number (%)*
Patient	15 (56)
Close person	12 (44)
**Patients (** ***n*** ** = 15)**	
**Gender** Female Male	7 (47) 8 (53)
**Age** 65–69 70–74 75–79 80–84 85–89 ≥ 90	2 (13) 1 (7) 2 (13) 5 (33) 4 (27) 1 (7)
**Study site** ^**#**^	
Site A	3 (20)
Site B	6 (40)
Site C	6 (40)
**eGFR (ml/min/1.73 m** ^**2**^ **)** < 10 10–14 ≥ 15	4 (27) 10 (67) 1 (7)^ø^
**Treatment plan** HD PD CKM Active on transplant waiting list	7 (47) 2 (13) 6 (40) 0 (0)
**Major comorbidities** Type 2 diabetes Ischaemic heart disease Hypertension Malignancy Obesity Heart failure Stroke Other comorbidity	10 (67) 7 (47) 5 (33) 4 (27) 2 (13) 1 (7) 1 (7) 5 (33)
**Cause of kidney disease** Type 2 diabetes Hypertension and/or vascular disease Removal of kidney cancer	10 (67) 3 (20) 2 (13)
**WHO performance status** 0 1 2 3 4	0 (0) 5 (33) 2 (13) 8 (53) 0 (0)
**Years of full time education** 0–5 6–10 11–15 16–20	1 (7) 6 (40) 6 (40) 2 (13)
**IMD** 1–2 3–4 5–6 7–8 9–10	4 (27) 3 (20) 6 (40) 1 (7) 1 (7)
**Number of family members recruited** 0 1 2	5 (33) 8 (53) 2 (13)
**Family members (** ***n*** ** = 12)**	
**Gender** Female Male	9 (75) 3 (25)
**Study site** ^**#**^	
Site A	2 (17)
Site B	4 (33)
Site C	6 (50)
**Relationship to patient** Spouse or partner Child Grandchild	6 (50) 5 (42) 1 (8)

* Due to rounding, percentages may not always appear to add up to 100%

#Site A: transplant centre with approximately 600 HD patients and a dedicated CKM clinic. Site B: transplant centre with approximately 600 HD patients and CKM provided through general nephrology care. Site C: non-transplant centre with approximately 200 HD patients and CKM provided through general nephrology care

øOne participant who had an eGFR of 25ml/min/1.73m^2^ at the time of interview, having been 14ml/min/1.73m^2^ at recruitment

Key. CKM, conservative kidney management; eGFR, estimated glomerular filtration rate; HD, haemodialysis; IMD, index of multiple deprivation; PD, peritoneal dialysis; WHO, World Health Organization

Interviews lasted 29 to 84 min. Illustrative transcript extracts are provided, with divergent views highlighted where relevant. Quotations are reported verbatim and marked with the participant group (patient/family member) and unique identifying number; age decade; gender (male, M; female, F); and clinically documented treatment plan (HD, PD or CKM). We did not observe noteworthy differences in perspectives between participants from the different recruiting centres, so have not labelled them thus.

Participants at various stages of kidney failure preparedness recounted their journey from diagnosis, generally marked by intense negative emotions, including shock and despair. Despite subsequent acceptance of kidney disease, an awareness of life’s fragility tended to have a persistent effect on individuals’ reflections upon their lives and futures. Whilst most accepted disease progression as inevitable, uncertainty lingered regarding prognosis; particularly relating to the tangled relationship between the prospect of death from kidney failure and competing risk of death from another cause. This interplay influenced perceptions of kidney function and treatment decisions:

*Maybe I will have to go on dialysis eventually*,* whether I will or not I don’t know… I mean not everybody goes on it I suppose*,* they’ve probably kicked the bucket before you get there… it’s in the lap of the gods really*,* isn’t it?**** #14 Patient (80s***,*** F***,*** PD)***

Here, we present three themes relating to participants’ understanding of kidney function and failure: (1) Kidneys as critical blood-cleaning organs, where kidney function was framed as vital for survival; (2) Unwitnessed function and failure, where disease was experienced invisibly; and (3) Quantifying and predicting kidney function; the conceptualisation of kidney function using numbers and graphs. Quotations exemplifying each theme are summarised in Table 2, with clinical implications.

**Table 2:** Illustrative quotations

Theme	Description and interpretation	Illustrative quotes	Clinical implications
1. Critical blood cleaning organs	Participants recognised kidneys as vital for sustaining life. Dialysis was understood to prolong life by substituting the kidneys’ blood cleaning function. Whilst mechanistic understanding of kidney and dialysis function was not erroneous, this reinforced the reductive concept that kidney failure necessitates dialysis, else death ensues. We explored this construct in further detail in another paper.(17)	*I“So if you’ve got kidney failure*,* that would be-* *P“That’s end of life virtually.”* **#3 Patient (80s**,** F**,** CKM)** *“Once again*,* my non-medical knowledge*,* your kidneys do a certain important job of straining out so many of the nasties from your body.”* **#1 Family member**,** wife of #1 patient (70s**,** M**,** PD)** *“It’s that [dialysis] or die basically. That’s the alternative*,* as far as I know of it.”* **#12 Patient (70s**,** M**,** HD)**	The expectation that to decline dialysis would be fatal may be seeded early in CKD care. Patients approaching kidney failure may be more able to choose the treatments that fit best with their values and preferences if clinicians can nuance their understanding and explanations. Communicating the following may help to temper the reductive idea of ‘do dialysis or die’. • Some people can live for many months or even years after reaching kidney failure (as defined by eGFR thresholds), without kidney replacement therapy. • Dialysis is always initiated before a person dies from kidney failure. This means that *all* recipients receive dialysis for a period during which they would be alive anyway. • Dialysis only ever provides a finite extension to life. • For those at the highest risk death from other causes – older people and those living with multiple comorbid illnesses –dialysis does not always extend life at all.
2. Unwitnessed function and failure	Many participants experienced advanced kidney disease as asymptomatic, reinforcing the concept that kidney function is either sufficient and compatible with life, or insufficient, indicating proximity of death. Invisibly reduced kidney function allowed dialysis decisions to be based upon blood test results, rather than upon experienced ill health and appeared to increase reliance upon information and guidance from clinicians.	*“I wouldn’t have been aware if nobody took these blood tests*,* I wouldn’t have been aware of it at all.”* **#3 Patient (80s**,** F**,** CKM)** *“It hasn’t been a visible problem at all*,* to either of us*,* and this is why I think we find it – Oooh – a bit of a shock when we suddenly get a note*,* a letter to say we must consider this [dialysis]”* *#***1 Family member**,** wife of #1 patient (70s**,** M**,** PD)** *“I just thought why would I want to be on dialysis when I was feeling so well?”* ***#14 Patient (80s***,*** F***,*** PD)***	Clinicians should be aware that the ‘unwitnessed’ nature of kidney disease could be disempowering, and seek ways of ensuring patients can still make person-centred decisions. One approach may be to routinely discuss and document the reasons for and goals of dialysis initiation – both in advance of, and at the time of, starting. Such would reinterpret the ‘need’ to start dialysis and could empower patients to share the decision as to whether biochemical or symptom indications are sufficient, for them, to indicate that the time to start dialysis has arrived.
3. Quantifying and predicting kidney function	Use of eGFR to quantify kidney function was ubiquitous. Misinterpretation appeared to impact upon person-centred decision-making regarding kidney failure treatment. Thresholds and ‘trigger levels‘ for dialysis initiation and death from kidney failure reinforced concrete associations between test results and necessitated clinical actions.	*“I think if you get to 15*,* they put you in for dialysis. That’s what I was told.”* **#4A Family member**,** daughter of #4 patient (80s**,** F**,** CKM)** *“Well (sighs) the 10’s the line*,* he told me 10. They like to start at 10.”* **#6 Patient (70s**,** F**,** HD)** *“They just showed me on a computer… this red line*,* they said*,* ‘This is your kidney function here and it’s up at 23%… and this is where your target*,* this is what’ll happen to you’”* **#10 Patient (80s**,** M**,** HD)**	No randomised controlled trial has ever identified an eGFR threshold below which careful monitoring for disease complications becomes unsafe. Clinicians should take care not to reinforce the ‘need’ for dialysis by establishing numerical thresholds. Patients and family members should be helped to understand that dialysis initiation decisions should not be governed by eGFR. Explaining the imprecision, variability, and extra-renal factors governing eGFR may help patients – and clinicians – to dissociate dialysis initiation decisions from eGFR thresholds. Alternative approaches – such as use of kidney failure risk equations – may be helpful.

### Theme 1: Critical blood-cleaning organs – “If your kidneys die, you’re dead.”

All participants appeared to recognise the kidneys as vital organs. This was explicit in statements linking kidney failure and death, but also seen in metaphors describing kidney failure as a “*death sentence”* (#10 patient) or “*the end of the road*” (#2 patient). Participants emphasised the blood-cleaning function of kidneys and dialysis, with few references to other functions. The only identified ‘cleaned’ substance was “potassium”. A wide variety of other negative, but abstract terminology was used including “*toxins*” (#10 patient, #12 patient, #6 patient*)*, “*poisons*” (#2 patient), “*nasties*” (#1 family member), “*impurities*” and “*naughty cells*” (#1 family member). #12 patient, who had been hospitalised with acute kidney injury, provided a detailed description of how “*toxin*” accumulation could be fatal:

*I don’t know which one goes first*,* your brain or your heart or whichever just dies of too high a level of toxin in your blood and that’s it*,* you slip into unconsciousness and you’re dead.**** #12 Patient (70s***,*** M***,*** HD)***

Patient participants appeared almost unanimous in their expectation that, if kidney failure arose, death from kidney failure would come, unless they died from a competing event (e.g. a “*car crash*” – #14 patient) or started dialysis. An assumption that dialysis would prolong life appeared across almost all accounts. The few participants who discussed death whilst in receipt of dialysis described this in terms of death from other causes; such as a *“heart attack”* (#1 patient), suggesting a widespread perception that dialysis prevents death from kidney failure. Participants had varied expectations about the experience of death without dialysis: some, especially those preparing for dialysis, anticipated death may be sudden and/or frightening, or else were uncertain what to expect; others, typically those planning for CKM, expected dying to be a less traumatic “sleep to the end” (#2 patient):

*…if you die from kidney failure you just go to sleep… which is quite pleasant…. You sort of die in your bed… it’s nothing to go against is it*,* another good point*,* another tick.**** #5 Patient (80s***,*** M***,*** CKM)***

Although numerous participants referred to prognostic discussions with clinicians, recollections of receiving a concrete prognosis were rare. This appeared to frustrate some participants who expressed a desire for more certainty:

*When we asked the doctor… ‘how long… we know you can’t say how long a piece of string is*,* but how long is his piece of string?’ (laughs)…. It’s sometimes worse not having all the facts.**** #8 Family member (wife of #18 patient***,*** 80s***,*** M***,*** HD)***

Where prognostic discussions were recalled, these generally related to death if dialysis was not initiated. Even those envisioning short remaining lives speculated about being compelled to start treatment, when faced with *“dialysis or death”* (#8 patient).

### Theme 2: Unwitnessed function and failure – “ The kidneys, I can’t see the kidneys…

Participants tended to depict kidney function as invisible. Some explained how they learned they had CKD from blood tests taken whilst feeling well, so the discovery came as a shock. Several participants articulated that the uncorroborated nature of low kidney function left them without an internal gauge of disease severity:

He [doctor] said ‘You’ll only know ‘cos you’ll be so tired, and you’ll have no energy- that’s the point you’ll know’. I haven’t experienced that yet so I cannot tell you. (laughs) **#1 patient (70s, M, PD)**

The asymptomatic and unwitnessed disease process appeared to allow some to consider their kidneys to be working “*properly*” (#11 patient). Other participants described how it was hard to accept having CKD, because they had never felt ill:

*You really don’t sort of believe that ‘cos I think because you feel perfectly alright so that’s it*,* they’re talking a lot of rot*,* but they obviously are not of course.**** #14 Patient (80s***,*** F***,*** PD)***.

Several participants, more commonly those preparing for dialysis, appeared uncertain about what problems to expect if their disease progressed, and whether their various symptoms could be attributed to their kidneys. Family members mentioned symptoms less frequently than patients, but when they did, appeared equally uncertain about what “*symptoms we’re to look out for*” (#4B family member). The invisible nature of kidney disease appeared to increase participants’ reliance upon the results of blood tests to guide their understanding of disease severity, and treatment decision-making.

### Theme 3: quantifying and predicting kidney function – “20% out of a hundred”

The use of eGFR to quantify kidney function was ubiquitous, despite no participant naming the term. Some referred to unitless numbers (nine participants); whilst 13 referred to the “*percentage for how much your kidneys are working*” (#15 patient).

More than half of participants discussed numerical ‘thresholds’ at which symptoms would develop, or, more commonly, “*trigger level[s]”* (#2 family member – a concept raised by 11 participants in total) at which someone would “*have to be on dialysis*” (#1 patient). Purported kidney function thresholds for dialysis initiation ranged between five (#10 patient) and 15 (#4A family member) and tended to be portrayed as concrete, rather than points of choice or discussion, since non-initiation of dialysis at this point appeared to be perceived as fatal. Whilst few appeared to consider thresholds for dialysis initiation as flexible, one participant recalled being informed of different cut-offs by different clinicians:

*…he thought that the level should be around 15*,* whereas the previous person had said 10 and below.**** #1 Family member (wife of #1 patient***,*** 70s***,*** M***,*** PD)***

Kidney function trends and “*scale[s]*” (#1 patient) appeared to contribute to perceptions such as “*stability*” (#4A and #4B family members), *“reversal”* (#15 patient) and *“steady decline”* (#1 patient):

*…we call it bobbing along the bottom of the pond because he goes up and down on the 20 line and he’s been doing that for a number of years now…* #8 Family member ***(wife of #8 patient***,*** 80s***,*** M***,*** HD)***

For some, these trends and scales appeared to have been consolidated by clinicians presenting graphs of kidney function results and extrapolating future trajectories. This practice appeared to emphasise the concept of relentlessly progressive disease and verified ‘trigger levels’ for both dialysis initiation and death from kidney failure – in many cases, considered to be one and the same:

… they said when you get down to 10 you ought to be on it and by 8 you’re definitely on it. ***#1 patient (70s, M, PD)***

Along with concrete thresholds for dialysis initiation, some participants (seven in total) appeared to understand that other factors may indicate that dialysis was indicated, or that – in the absence of dialysis – symptoms indicated that the end of life was near. Several appeared uncertain about the relative contribution of numerical kidney disease measures to their future health in the context of their comorbidities and ageing:

*…how long can you live on 12%? I don’t know.**** #5 Patient (80s***,*** M***,*** CKM)***

Another planning for HD (#11 patient) discussed how dialysis may be started if his “*health*” was “*going down*”. One participant in receipt of CKM (#2 patient) saw his progressive “sleepiness” as evidence that death from kidney failure was nigh:

*I don’t know when it’s [dialysis] going to start or nothing… Well*,* if my health’s going down or something like that*,* it’s totally up to my doctor… It’s entirely up to my health**** #11 patient (80s, M, HD)***

*What it says in the books is quite correct so far as sleepiness is concerned. It’s going on and on and on*,* and it’s becoming progressively greater*,* as it were*,* as time goes on. ****#2 patient (90s, M, CKM)***

Despite this, participants who discussed non-numerical reasons for dialysis initiation continued to express that numerical thresholds and clinician-driven decisions may be the primary indicators, with five of the seven referencing numerical estimates of kidney function, including three as “*percentage*” function. Overall, patient and family member participants alike appeared unclear about indications for dialysis initiation, which appeared to accentuate reliance upon the tangible information provided by eGFR.

## Discussion

Elsewhere, we have published analysis from this work of

i) Patient interviews, focussing on participants’ perceptions of the decision between, and prognostic implications of dialysis and CKM [17]. We found that unfamiliarity with treatment options, the desire to live, and a ‘do or die’ notion conspire to cast haemodialysis as inevitable; and.
ii) Patient-carer dyadic data, exploring familial perspectives on kidney failure treatment decision-making [18]. We found that family members have significant influence on older patients’ kidney failure treatment decisions, in addition to the information and support provided by clinicians through shared decision-making support.

In this new analysis we have identified misperceptions about CKD and kidney failure which may impact upon older patients’ and family members’ ability to make informed treatment decisions between dialysis and CKM. The discourse of advanced kidney disease appears to be similarly perceived by patients and family members, primarily through; (a) concepts of ‘toxin’ clearance; and (b) low eGFR values, often conveyed in the language of ‘percentage kidney function’. These constructs are developed in the context of an ‘invisible’ illness experience and uncertainties regarding the interconnectedness of kidney failure with concurrent health conditions and overall prognosis, which may render patients susceptible to treatment initiation based on test results, rather than person-centred reasoning [7]. Misperceptions of a “need” for dialysis initiation upon reaching numerical thresholds based on inaccurate expectations or reasons for starting may undermine older patients’ and their families’ capacity to envision a future living without dialysis, particularly if dialysis has been presented as the superior treatment option [29, 30]. This has implications for future approaches to CKD education which should involve both older patients and their family members.

Whilst the concepts of ‘toxin’ clearance and kidney failure as indicated by reduced eGFR may be factually correct, and likely reflect clinical communication and constructs, they drive assumptions which present a potentially major impediment to shared decision-making. The reductive and mechanistic model of dialysis being “needed” once a threshold kidney function is reached seems especially problematic. This appears to undermine the acceptability of delayed dialysis initiation, or conservative kidney management, as treatments that can be reflective of people’s priorities and goals, in terms of acceptable survival, quality of life outcomes, and the nature and quality of the end of their lives [31]. This may be especially true for older people and those with multiple long-term conditions, for whom the survival and quality of life benefits of kidney replacement therapy are least certain [4]. 

Studies of younger people with CKD have previously shown how knowledge gaps and misunderstandings can impact on health behaviours, such as self-management, medication concordance, and perceptions about risks of progression to kidney failure [32]. While other studies have explored treatment decision making processes among older patients with CKD [33, 34], our study is one of few [12] to specifically explore the impact of disease understanding on treatment decisions made by this patient group. The important nuances of the decisions they face did not appear to have been conveyed: as we described previously in a separate paper examining patient perceptions of dialysis decision making [17], there was little evidence in the current study that older patients, many with multiple long-term conditions, or their family members, understood that dialysis might not prolong their lives [35].

Our analysis suggests that the often ‘invisible’ experience of advanced kidney disease can render numerical measures and trends more able to dominate perceptions of future health. Amongst our participants, the belief that they must “do dialysis or die” [17]– which has been described before [34, 36–38] – appeared to be driven in part by perceptions of percentage function as absolute, future decline along a scale as relentlessly progressive, and existence of numerical trigger levels. Together, these established the idea that a “*need*” to initiate dialysis treatment, or face death, was inevitable once kidney function fell below a certain threshold.

The ‘unwitnessed’ nature of kidney disease appeared to increase reliance upon information and guidance from clinicians. That CKD can be experienced as ‘unwitnessed’ has been described across age-groups [12, 36, 39, 40]. Feelings of uncertainty, disconnectedness and lack of control have been described previously in CKD [36, 39, 41, 42] and other ‘unwitnessed’ conditions such as chronic leukaemia [43]. The gap left by intangibility appears readily filled by blood test results: eGFR governed older patient and family understanding of disease, treatment decisions, and expectations for the future. This has been observed before amongst older patients with CKD [36, 41]; however earlier studies did not involve family members, known to play an important role in shared decision-making [15, 44], nor have they demonstrated previously how perceptions, and misperceptions, of these numerical measures might influence kidney failure treatment decisions in this older patient group.

An expectation that dialysis will be ‘needed’, and that to decline it would be fatal, may arise early during CKD care. Clinicians should take care not to reinforce this ‘need’ by establishing numerical thresholds. It is well-established that failure to adequately communicate disease information, including numerical information, negatively impacts shared decision-making, empowerment, and self-management [45–47]. Low health literacy, including numeracy, common among many groups of people with CKD [48, 49] and older people [50], can also contribute to misinterpretation of health risks [45, 51]. Whilst thresholds and trends in eGFR play a role in CKD care pathways and disease staging [52, 53], trigger levels are not supported by clinical research. No randomised controlled trial has ever identified a threshold below which careful monitoring for disease complications becomes unsafe [54]. Despite being technically inaccurate [55], the shorthand “percentage” kidney function is likely intended to aid patient understanding. Like others [39] we have found that the concept appears difficult for older patients and family members to interpret. Amongst our older patient and family member group, there was no mention of imprecision in estimated kidney function [56]; of alternative explanations for “stable” or rising eGFR, such as sarcopenia; or of the possibility of abrupt decline to kidney failure [57]. 

Defining the reasons and optimal timing for dialysis initiation for older patients is challenging, requiring consideration of individual patients’ circumstances and preferences. Potential benefits of earlier treatment initiation, such as initiation of chosen modality, must be weighed against the potential for receipt of dialysis that provides neither quality nor quantity of life benefit [58]. A focus on eGFR may compel older patients to prepare for dialysis without consideration of wider factors which can have a significant personal impact, such as symptoms, quality of life, treatment burden and end-of-life wishes.

Optimal communication depends on clinicians having skills and resources to facilitate complex discussions; however nephrologists describe challenges explaining disease, treatment and prognostic complexity within available consultation time [7, 12, 31, 36, 39], and currently available patient information documents may misrepresent treatment options [59]. One approach may be for clinicians to routinely discuss with patients and document the goals of dialysis initiation – both in advance of, and at the time of, starting. Such would reframe the ‘need’ as the ‘reason’ to start dialysis. This might empower patients to share the decision about whether such a time has come, and to judge whether initiation has been successful. It may also help patients – and clinicians – to nuance understanding of the associations between kidney failure (as defined by eGFR thresholds), kidney replacement therapy, and death. People can live for many months or even years after reaching kidney failure, without kidney replacement therapy [60]. Dialysis only ever provides a finite extension to life, and must be started before death from kidney failure. Older people, and others at high risk of competing mortality need accurate, unbiased information which conveys the reality that life extension cannot be presumed with dialysis initiation, in conjunction with compassionate and supportive input from their clinical teams [4]. Research involving consultation observation may help to identify how communication influences patient understanding and decision-making, and inform approaches that could be included in interventions [29]. 

The strengths of this study include a rigorous application of qualitative methods and broad range of clinicodemographic variation between study participants, sampled from multiple kidney centres. The inclusion of family members brings in the views of an often neglected group, known to play an important role in treatment decision-making [15, 44]. The study has limitations. All centres were in England and all participants were white British. Culture and faith play important roles in understandings of disease and treatment decision-making, so our findings may not be transferable to non-white British patient groups. Further research with ethnically diverse groups is needed. Patient participants had a range of educational levels, with socioeconomic backgrounds skewed towards lower levels of deprivation. Experiences and understandings may differ among patients from different socioeconomic backgrounds. The lack of noteworthy differences in the perspectives of participants from recruiting centres selected for variation in CKM models could be due to the small sample size in this qualitative study. It might also reflect that much clinical communication and acquisition of disease understanding pre-dates treatment decision-making and kidney failure management. All findings are based on patient and caregivers’ personal experiences: direct observation of how kidney function and failure are described in clinical practice would be informative [29, 59]. As with all qualitative research, the authors’ own experiences will have influenced data and analysis. As trainees in qualitative research, BH, CS and RK’s skills were developing and this may have affected the depth and breadth of findings, though this was mitigated by an experienced supervisory team. Finally, data collection was discontinued when analysis of patient interviews indicated sufficient informational power, so themes from family member interviews may have been less well developed.

## Conclusion

In conclusion, we found older patients with advanced CKD and their family members held similar, simplified constructs of kidney function and failure. Whilst similar misperceptions have been demonstrated amongst younger people with kidney disease, our study identifies the unique implications of these where older individuals face a decision between dialysis and CKM. Current approaches to CKD education may be unsuitable for this older patient group and their families, for whom decisions about preparation for kidney failure are especially preference-sensitive. Evidence-based guidance and clinician education are needed to ensure information about kidney failure and its treatments is effectively communicated. Revising inaccurate and misleading terminology may help patients and families to make better informed and holistic choices about their future priorities and treatment preferences. The recently advocated move towards use of risk-based equations for guiding CKD referral and treatment decisions [61, 62] could provide an opportunity to re-examine how best to communicate disease severity in CKD, and its implications for treatment decisions, to older patients and their families. Improvement requires the development of evidence-based communication guidance, training and education, decision aids, and health literacy assessment; adapted for specific patient groups, all of which have the potential to enhance shared decision-making, advance care planning, and patient-centred outcomes [7, 12, 63]. Our findings suggest that clinicians should shift focus away from numerical measures of disease severity towards wider factors guiding treatment decisions, such as quality of life, individualised values and goals. This may help people living with advanced kidney disease to better trade-off the benefits and burdens of available treatments.

## Supplementary Information

Below is the link to the electronic supplementary material.

Supplementary Material 1

Supplementary Material 2

Supplementary Material 3

Supplementary Material 4

## Data Availability

Raw interview data is potentially identifiable so is not made available. Please contact the corresponding author for further details.

## Abbreviations

CKD: Chronic kidney disease
KRT: Kidney replacement therapy
CKM: Conservative kidney management
eGFR: Estimated glomerular filtration rate
HD: Haemodialysis, MLTC–multiple long–term conditions
PD: Peritoneal dialysis
SDM: Shared decision making
