# Supplementary figures and images for: How do older patients with advanced kidney disease, and their family members, understand kidney function and failure? A qualitative study
Source: BMC Nephrol. 2025 Nov 4;26:613. doi: 10.1186/s12882-025-04541-1 (PMC12584381; doi:10.1186/s12882-025-04541-1)

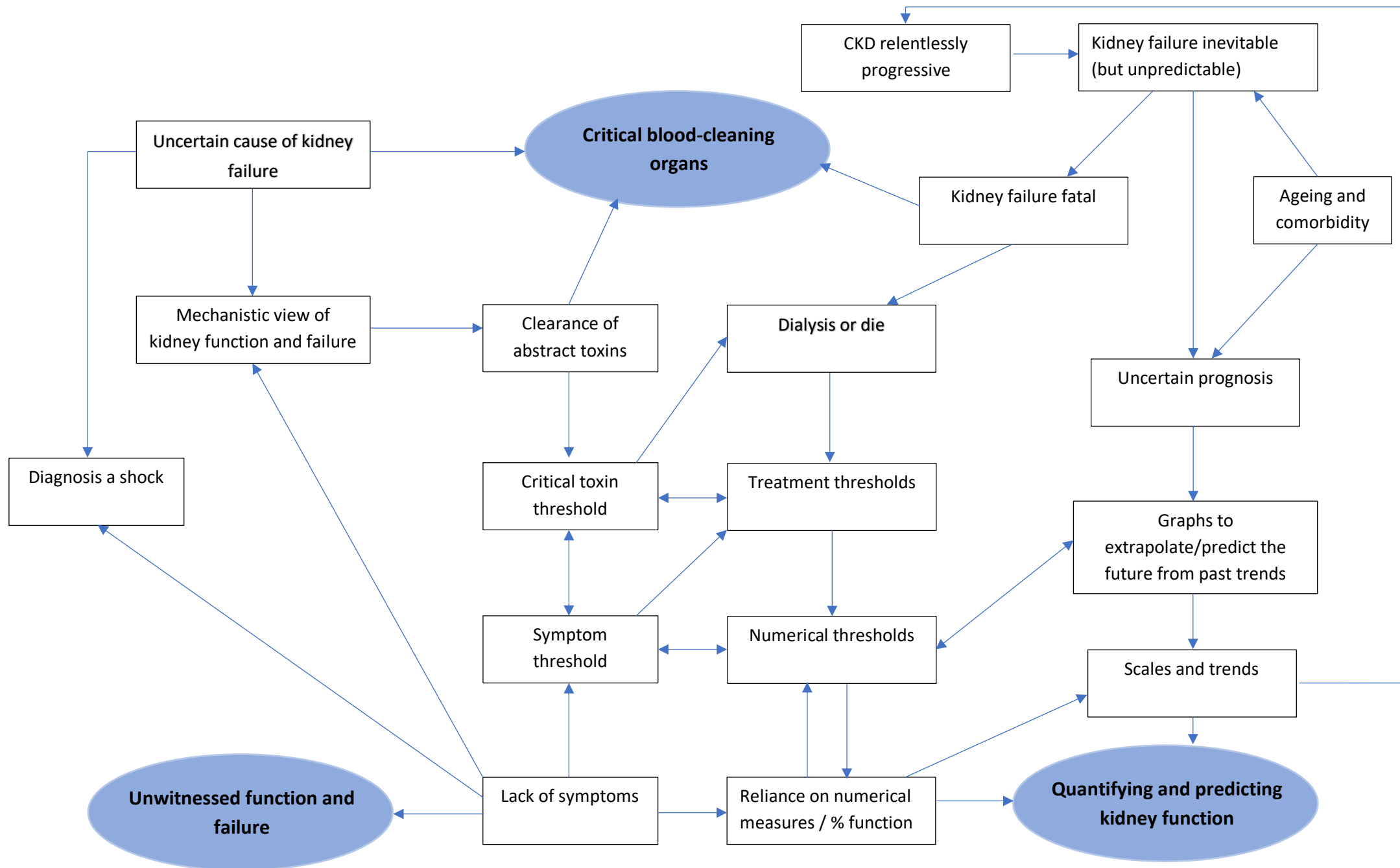

**Supplementary File S3.** Thematic map linking sub-themes into broader overarching parent themes

Supplement: Supplementary file 3 — Supplementary Material 3 [file 12882_2025_4541_MOESM3_ESM.pdf]

**Supplementary File S4.** Study flow diagram

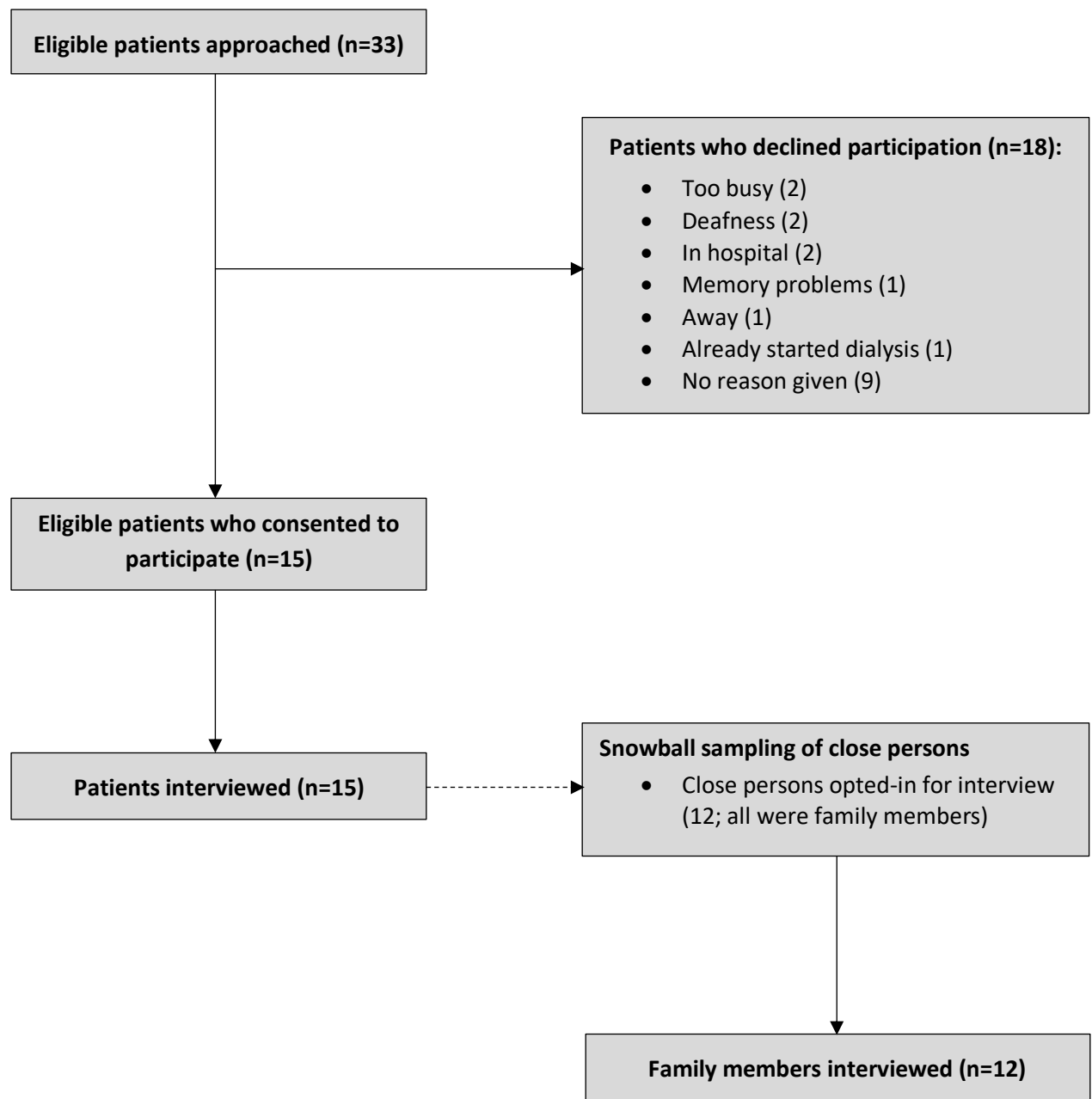

Supplement: Supplementary file 4 — Supplementary Material 4 [file 12882_2025_4541_MOESM4_ESM.pdf]
